# The Transcriptional Repressor PerR Senses Sulfane Sulfur by Cysteine Persulfidation at the Structural Zn^2+^ Site in *Synechococcus* sp. PCC7002

**DOI:** 10.3390/antiox12020423

**Published:** 2023-02-09

**Authors:** Daixi Liu, Hui Song, Yuanning Li, Ranran Huang, Hongyue Liu, Kunxian Tang, Nianzhi Jiao, Jihua Liu

**Affiliations:** 1 Institute of Marine Science and Technology, Shandong University, Qingdao 266237, China; 2 School of Pharmaceutical Sciences, Shandong University, Jinan 250012, China; 3 Joint Lab for Ocean Research and Education at Dalhousie University, Shandong University and Xiamen University, Qingdao 266237, China; 4 Third Institute of Oceanography, Ministry of Natural Resources, Xiamen 361000, China

**Keywords:** cyanobacteria, sulfane sulfur, PerR, peroxiredoxin, transcriptional regulator

## Abstract

Cyanobacteria can perform both anoxygenic and oxygenic photosynthesis, a characteristic which ensured that these organisms were crucial in the evolution of the early Earth and the biosphere. Reactive oxygen species (ROS) produced in oxygenic photosynthesis and reactive sulfur species (RSS) produced in anoxygenic photosynthesis are closely related to intracellular redox equilibrium. ROS comprise superoxide anion (O_2_^●−^), hydrogen peroxide (H_2_O_2_), and hydroxyl radicals (^●^OH). RSS comprise H_2_S and sulfane sulfur (persulfide, polysulfide, and S_8_). Although the sensing mechanism for ROS in cyanobacteria has been explored, that of RSS has not been elucidated. Here, we studied the function of the transcriptional repressor PerR in RSS sensing in *Synechococcus* sp. PCC7002 (PCC7002). PerR was previously reported to sense ROS; however, our results revealed that it also participated in RSS sensing. PerR repressed the expression of *prxI* and downregulated the tolerance of PCC7002 to polysulfide (H_2_S_n_). The reporter system indicated that PerR sensed H_2_S_n_. Cys^121^ of the Cys4:Zn^2+^ site, which contains four cysteines (Cys^121^, Cys^124^, Cys^160^, and Cys^163^) bound to one zinc atom, could be modified by H_2_S_n_ to Cys^121^-SSH, as a result of which the zinc atom was released from the site. Moreover, Cys^19^ could also be modified by polysulfide to Cys^19^-SSH. Thus, our results reveal that PerR, a representative of the Cys_4_ zinc finger proteins, senses H_2_S_n_. Our findings provide a new perspective to explore the adaptation strategy of cyanobacteria in Proterozoic and contemporary sulfurization oceans.

## 1. Introduction

The environment on Earth transformed from anaerobic to aerobic during the evolution of life [1]. Cyanobacteria, some of the oldest microorganisms on Earth that can perform both anoxygenic and oxygenic photosynthesis, were a key driving force during evolution [2]. Life is created, regulated, and sustained by reduction–oxidation (redox) reactions, and ROS and RSS are two critical kinds of signal intracellular molecules associated with the redox balance [3]. In Proterozoic oceans, cyanobacteria perform anoxygenic photosynthesis, using alternative reduced electron donors, such as hydrogen sulfide (H_2_S) [4]. Therefore, RSS should be the major participant in the regulation of the intracellular redox balance in cyanobacteria. Oxygenic photosynthesis, which uses solar energy to pry electrons from water, became a major part of the Earth’s ecosystems as it succeeded in oxygenating the atmosphere and the biosphere more than 3 billon years (Ga) ago [5]. The main player in redox regulation became ROS. Although the modern ocean is aerobic, there are still some areas that lack oxygen, such as oxygen-minimum zones [6] and microbial mats [7]. The versatility of cyanobacteria to perform both anoxygenic photosynthesis and oxygenic photosynthesis has therefore been conserved [8], with cyanobacteria coping with both ROS and RSS in living environments. The metabolic regulation mechanism of ROS in cyanobacteria has widely been reported [9,10,11]; however, the sensing mechanisms of RSS have remained incompletely understood.

RSS, similar to ROS, are important cellular signaling molecules [3]. ROS comprise O^2●−^, H_2_O_2_, and hydroxyl radicals (^●^OH), which are products of molecular oxygen accepting electrons from cellular redox components [12]. H_2_S and sulfane sulfur are representatives of RSS, which are produced during the process of sulfur-containing compound metabolism [13,14,15]. Even though H_2_S was originally thought to be a gasotransmitter [16], emerging evidence suggests that sulfane sulfur plays a more important role in signal transduction [17,18,19,20]. Sulfane sulfur consists of various forms of zero-valent sulfur, including persulfide forms (RSSH and HSSH), polysulfide forms (RSS_n_H, RSS_n_R, and H_2_S_n_, *n* ≥ 2), and elemental sulfur (S_8_). Sulfane sulfur operates via a similar mechanism to ROS while acting as a signaling molecule, namely, by modifying protein at a cysteine residue. In addition, the second and equally important mechanism would be performed by reacting with metal centers in proteins [21]. S-sulfhydration could protect cysteine residues from ROS-mediated damaging oxidation [22]. However, excess sulfane sulfur can initiate complex antioxidant reactions, even affecting cellular processes, which is catastrophic for cellular function [23]. Furthermore, sulfane sulfur participates in the regulation of gene expression in photosynthesis [24]. Therefore, it is important to maintain intracellular sulfane sulfur homeostasis.

Microorganisms have evolved a series of protective enzymes to maintain intracellular sulfane sulfur concentration within safe limits. Sulfide:quinone oxidoreductase (SQR) [15], persulfide dioxygenase (PDO) [25], and flavocytochrome c sulfide dehydrogenase (FCSD) [26] are involved in the process. Under normal conditions, SQR oxidizes H_2_S and produces sulfane sulfur, which is further oxidized by PDO. FCSD is another type of enzyme that also oxidizes H_2_S. However, some ROS coping strategies were also reported to be suitable for sulfane sulfur, such as superoxide dismutases (SOD), catalase, thioredoxin (Trx), glutaredoxin (Grx), and peroxiredoxin (Prx). SOD was reported to metabolize H_2_S and produce RSS, and catalase could also act as a H_2_S oxidase [27,28,29]. Trx, Grx and Prx systems also participate in the process of sulfane sulfur reduction [30,31]. Thus, there is a close relationship between RSS and ROS metabolism.

The response to sulfane sulfur in bacteria is often coordinated by transcription factors [32]. CstR [33], BigR [34], SqrR [35], and FisR [36] are major transcription factors. CstR, BigR, and SqrR are transcription repressors, negatively regulating the transcription of H_2_S oxidation-related genes, while FisR is a σ^54^-dependent transcription activator. These regulatory factors display a similar mechanism of RSS sensing; that is, RSS modifies the two cysteines on the protein to form a Cys-S-S-Cys structure. OxyR was also reported to participate in sulfane sulfur sensing [30]. *Escherichia coli* OxyR was the earliest transcription factor discovered; it regulates the expression of *katG* (encoding catalase), *trxC* (encoding Trx), and *grxA* (encoding Grx). OxyR is a transcriptional activator that acts via the formation of a disulfide bond between the C^199^ and C^208^ residues, while sensing H_2_O_2_ [37]. Sulfane sulfur modifies OxyR at Cys^199^ and forms a persulfide OxyR Cys^199^-SSH, thus activating the expression of the *trx* and *grx* genes. PerR is another type of peroxide-sensing regulator, which is complementary to OxyR; therefore, these two regulators do not usually exist in the same bacterium. PerR, which belongs to the Fur family, is a metal-dependent regulator that represses the expression of oxidative stress genes (*prx* and *ahpc*) [38,39]. PerR contains a DNA-binding region, a Zn^2+^-binding site consisting of cysteine residues, and a Fe^2+^/Mn^2+^-binding site consisting of histidine and aspartic acid residues. Based on the above, we deduced that PerR may also be involved in sulfane sulfur sensing, but the mechanism may be different from that of OxyR. This hypothesis remains to be explored.

Many studies have focused on the role of PerR in H_2_O_2_ sensing, but the mechanism of sulfane sulfur sensing is still unclear. Li et al. found that PerR in *Synechocystis* sp. PCC6803 binds to the promoter region of *prx* to regulate its expression in response to peroxide stress [9]. Ludwig et al. found that *prx* expression in PCC7002 was also regulated by PerR [40]. However, these studies did not resolve the specific regulation mechanism of PerR. The mechanism by which PerR senses H_2_O_2_ in *Bacillus subtilis* has been reported in detail. *B. subtilis* PerR contains a structural metal ion (Zn^2+^) binding site and a regulatory metal ion (Fe^2+^ or Mn^2+^) binding site. In the presence of excess H_2_O_2_ or O_2_, the two histidines that constitute the binding site of regulatory metal ions are oxidized, and the inhibitory effect of PerR is released. This is the main mechanism by which PerR senses H_2_O_2_ [38]. In addition, four conserved cysteines combine with Zn^2+^ to form a Cys_4_:Zn^2+^ structure, which also plays a key role in the process of redox regulation [39]. Based on this mechanism of H_2_O_2_ sensing and considering that the site of action of sulfane sulfur is cysteine [41], we speculated that the active site of sulfane sulfur on PerR may be the Cys_4_:Zn^2+^ structure. It has not been reported how, or indeed whether, the Cys_4_:Zn^2+^ structure is affected by sulfane sulfur, and thus, the mechanism needs to be explored in greater depth.

Here, we report that *Synechococcus* PerR senses sulfane sulfur and regulates the expression of *prxI*. PerR effectively decreases the tolerance of PCC7002 to sulfane sulfur by altering the expression of *prxI*. Sulfane sulfur modifies Cys^19^ and Cys^121^ to form Cys^19^-SSH and Cys^121^-SSH; as a result, the zinc atom is released from the Cys_4_:Zn^2+^ site, destroying the function of PerR. The discovery that sulfane sulfur acts on the Cys_4_:Zn^2+^ site of regulators is significant. Our findings reveal a new sulfane sulfur sensing mechanism, and provide a new perspective for exploring the adaptive mechanism of cyanobacteria in the evolution from an anaerobic environment to an aerobic one on Earth and the contemporary anoxic environment.

## 2. Materials and Methods

### 2.1. Strains and Culture Conditions

PCC7002 and its mutants (PCC7002Δ*perR* and PCC7002Δ*prxI*Δ*perR*) were grown in conical flasks containing medium A^+^ [42] under continuous illumination of 50 μmol photons m^−2^·s^−1^, at 30 °C. To sustain normal growth, 30 µg/mL chloramphenicol was added to the medium of PCC7002Δ*perR*, and 50 µg/mL kanamycin and 30 µg/mL chloramphenicol were added to the medium of PCC7002Δ*prxI*Δ*perR*. *Escherichia coli* strains were cultured in LB medium, at 37 °C. All strains and plasmids are listed in Table S1.

### 2.2. Construction of PCC7002 Mutants

The PCC7002Δ*prxI* mutant was constructed in our previous study [31]. PCC7002Δ*perR* and PCC7002Δ*prxI*Δ*perR* were constructed by natural transformation and homologous recombination according to a previously reported method [24]. The plasmid used in *perR* deletion was constructed as follows. First, two segments, ~1000-bp long, immediately upstream and downstream of the *perR* gene, were acquired using the primer sets *perR*-del-1/*perR*-del-2 and *perR*-del-5/*perR*-del-6 (Table S2) by PCR from genomic DNA of PCC7002. The chloramphenicol resistance cartridge was amplified with the primers *perR*-del-3/*perR*-del-4. Second, the above three segments were fused by PCR, and they were connected with the pJET1.2 blunt vector by the TEDA method [43]. Then, the product was transformed into *E. coli* DH5α by electroporation, and correct transformants were verified by PCR and sequencing. For PCC7002Δ*perR*, the correct plasmid was transformed into PCC7002 by natural transformation. For PCC7002Δ*prxI*Δ*perR*, the correct plasmid was transformed into PCC7002Δ*prxI*. Here, 30 µg/mL chloramphenicol was used to select for correct transformants. Finally, the mutants PCC7002Δ*perR* and PCC7002Δ*prxI*Δ*perR* were verified by PCR and sequencing. 

### 2.3. The Toxicity of H_2_S_n_ against PCC7002, PCC7002ΔperR, and PCC7002ΔprxIΔperR

H_2_S_n_, at concentrations of 1, 3, and 5 mM, was added to the sealed centrifugation tubes containing PCC7002, PCC7002Δ*perR*, and PCC7002Δ*prxI*Δ*perR* cells at log phase with an OD_730nm_ of 0.6–0.7. H_2_S_n_ was prepared according to a previously reported method with minor modification [15]. Briefly, sulfur powder, NaOH, and NaHS were mixed in a 1:1:1 molar ratio and dissolved in distilled water under argon gas in sealed bottle. Then, the bottle was incubated, at 37 °C, till sulfur was completely dissolved. After 6 h incubation, at 30 °C, under continuous illumination of 50 μmol photons·m^−2^·s^−1^, cells were washed and resuspended in fresh A^+^ medium. Then, 10 µL of cells was placed on the A^+^ agar plate after diluting with A^+^ medium to 10^0^, 10^−1^, and 10^−2^. The plates were cultivated at 30 °C under continuous illumination of 50 μmol photons·m^−2^·s^−1^ for 7 days.

### 2.4. Induction, RNA Extraction, and qRT-PCR Analysis

PCC7002 and PCC7002Δ*perR* cells at log phase with an OD_730 nm_ of 0.6–0.7 were incubated with or without H_2_S and H_2_S_n_ (at concentrations of 250 and 500 µM) in sealed centrifuge tubes for 3 h, at 30 °C, and 50 μmol photons·m^−2^·s^−1^ illumination. H_2_S was prepared according to the previous report [44] and experimental requirements, and the preparation method was as follows: 56.06 mg NaHS was dissolved into 1 mL of buffer (containing 50 mMTris.HCL and 100 μM DTPA), which had been degassed with N_2_ prior to NaHS powder solubilization, and diluted according to the desired concentration. Then, the induced cells were harvested by centrifugation at 10,000× *g*, and 4 °C for 10 min. Total RNA was isolated using the TaKaRa MiniBEST Universal RNA Extraction Kit, and the concentration and quality of RNA were verified by Qubit 4 (Invitrogen, Carlsbad, CA, USA). The cDNA was acquired using the Prime Script™ RT reagent kit with gDNA Eraser (TaKaRa, Dalian, China). qRT-PCR was performed using the CFX96 Touch Real-Time PCR Detection System (Bio-Rad, Hercules, CA, USA) with the SYBR^®^ Premix Ex Taq™ II kit (TaKaRa, Dalian, China). The primers used for the target genes are shown in Table S1. The reference gene *rnpA* (SYNPCC7002_A0989) was also included [45]. The results were analyzed according to the 2^−ΔΔCT^ method [46].

### 2.5. Construction of the perR-Repressed Reporter System

A perR-repressed reporter system in *E. coli* BL21 was constructed to assess the regulatory role of PerR on *prxI* expression. The plasmid pBBR-*perR*-Pp*rxI*-*egfp* was constructed as follows: The *perR* gene was expressed under the control of the *lacI* promoter, and the *egfp* gene was expressed under the control of the *prxI* promoter. PerR could act on the promoter region of *prxI*, thus influencing the fluorescence of GFP. The effect of H_2_S_n_ and S_8_ on PerR were evaluated by the changes in fluorescence intensities. The plasmid was transformed to *E. coli* BL21 for further study. *E. coli* BL21 (pBBR-*perR*-P*prxI*-*egfp*) was cultured in LB media, at 37 °C, to logarithmic phase (OD_600nm_ = 0.6) and 0.5 mM of isopropyl β-D-thiogalactoside (IPTG) was added to induce *PerR* expression. Then, H_2_S, H_2_S_n_, and S_8_ (at concentrations of 0, 150, and 300 µM) were added to the medium and the cells were cultured for another 2 h. Finally, the cells were collected and washed twice with 50 mM PBS (pH 7.4) to detect the fluorescence of GFP at excitation and emission wavelengths of 482 nm and 515 nm.

The six cysteines of PerR in the pBBR-*perR*-P*prxI*-*egfp* plasmid were all mutated to serines using the primer pairs PerR-C19S-F/R, PerR-C121S-F/R, PerR-C124S-F/R, PerR-C137S-F/R, PerR-C160S-F/R, and PerR-C163S-F/R by a modified QuickChange Site-Directed Mutagenesis Method [47]. *E. coli* BL21 (pBBR-*perR*_C-S_-P*prxI*-*egfp*) cells at logarithmic phase were induced with 0.5 mM IPTG and incubated with 300 µM H_2_S_n_ for 2 h to investigate the role of cysteines in PerR.

### 2.6. Construction, Overexpression, and Purification of PerR

PerR was fused to the C-terminus of maltose binding protein (MBP) and overexpressed in the vector pMal-C2X [48]. To achieve this, the perR fragment was amplified from the PCC7002 genome using the primer pair pMal-*perR*-F/R, ligated to pMal-C2X by the TEDA method, and transformed into *E. coli* DH5α. Verified plasmid was then transformed into *E. coli* BL21(DE3), and the resulting pMal-*perR* cells were cultured in LB medium, at 37 °C, to an OD_600nm_ of 0.6. Then, 0.5 mM IPTG was added for an additional 6 h incubation, at 30 °C. The cells were disrupted by a pressure cell homogenizer (SPCH-18; Stansted Fluid Power Ltd., London, UK). The cell debris was removed by centrifugation at 13,000× *g* and 4 °C for 20 min. PerR protein with the MBP (MBP-PerR) was separated by Amylose Resin Column (Invitrogen, Carlsbad, CA, USA) according to the supplier’s recommendations. PerR was released from the fusion with MBP using Factor Xa, at room temperature, for 24 h.

### 2.7. Zn^2+^ Release Assay

PAR could bind to Zn^2+^ and the Zn^2+^-PAR complex had maximum absorption at 494 nm; thus, absorption was used to indicate the amount of Zn^2+^. Here, 5 µM of purified PerR was treated with 10 mM H_2_S_n_ or 10 mM H_2_O_2_ in the presence of 100 µM PAR at 25 °C, and released Zn^2+^ ions were measured by monitoring the Zn^2+^-PAR complex at 494 nm every 1 s for 10 min. PerR without treatment was used as a control.

### 2.8. LC-MS/MS Analysis of PerR

Purified PerR at 5 mg/mL was reacted with 1 mM H_2_S_n_ sulfur or DTT for 30 min, at 25 °C. The reacted protein was treated with denaturing buffer (0.5 M Tris-HCl, 2.75 mM EDTA, 6 M guanadine-HCl, pH 8.0), then incubated with 1 M iodoacetamide (IAM) for 1 h in the dark. The sample was subsequently digested with trypsin (1:25, *w*/*w*), at 37 °C, for 20 h and subjected to C18 Zip-Tip (Millipore, Burlington, MA, USA) purification for desalting before analysis by HPLC-tandem mass spectrometry (LC-MS). A gradient of solvent A (0.1% formic acid in 2% acetonitrile) and solvent B (0.1% formic acid in 98% acetonitrile) from 0% to 100% in 100 min was used for elution in the Prominence nano-LC system (Shimadzu, Kyoto, Japan). LTQ-OrbitrapVelos Pro CID mass spectrometer (Thermo Scientific, Waltham, MA, USA) was used to ionize and electrospray the eluent, which was run in data-dependent acquisition mode with Xcalibur 2.2.0 software (Thermo Scientific, Waltham, MA, USA). Fullscan MS spectra (from 400 to 1800 *m*/*z*) were detected in the Orbitrap with a resolution of 60,000 at 400 *m*/*z* [17,36,49,50,51,52].

### 2.9. Phylogenetic Analysis

Cyanobacterial genomes were downloaded from the NCBI database. The sequences in Table S3 were used as queries to obtain PerR candidates. The candidates were obtained by searching the database with the standalone BLASTP algorithm, using conventional criteria (E value of ≥1 × 10^−5^ coverage of ≥45%, and identity of ≥30%) [53]. PerR candidates were aligned using MAFFT version 7.490 [54] with the option “-auto-maxiterate 1000”, and ambiguously aligned regions were removed using trimAl version 1.4 [55] with the “gappyout” option. Phylogenetic analysis was performed based on maximum likelihood methods using IQ-TREE [56] with automatic detection of the best-fit model with the “-MFP” option using ModelFinder [57] under the Bayesian information criterion (BIC). The topological robustness of the tree was evaluated by 1000 ultrafast bootstrap replicates. PerR proteins from *Staphylococcus epidermidis*, *Staphylococcus haemolyticus*, and *Staphylococcus aureus*, detailed in Table S3 were used as an outgroup.

## 3. Results

### 3.1. Phylogenetic Analysis of PerR in Cyanobacteria

**Figure 1:**
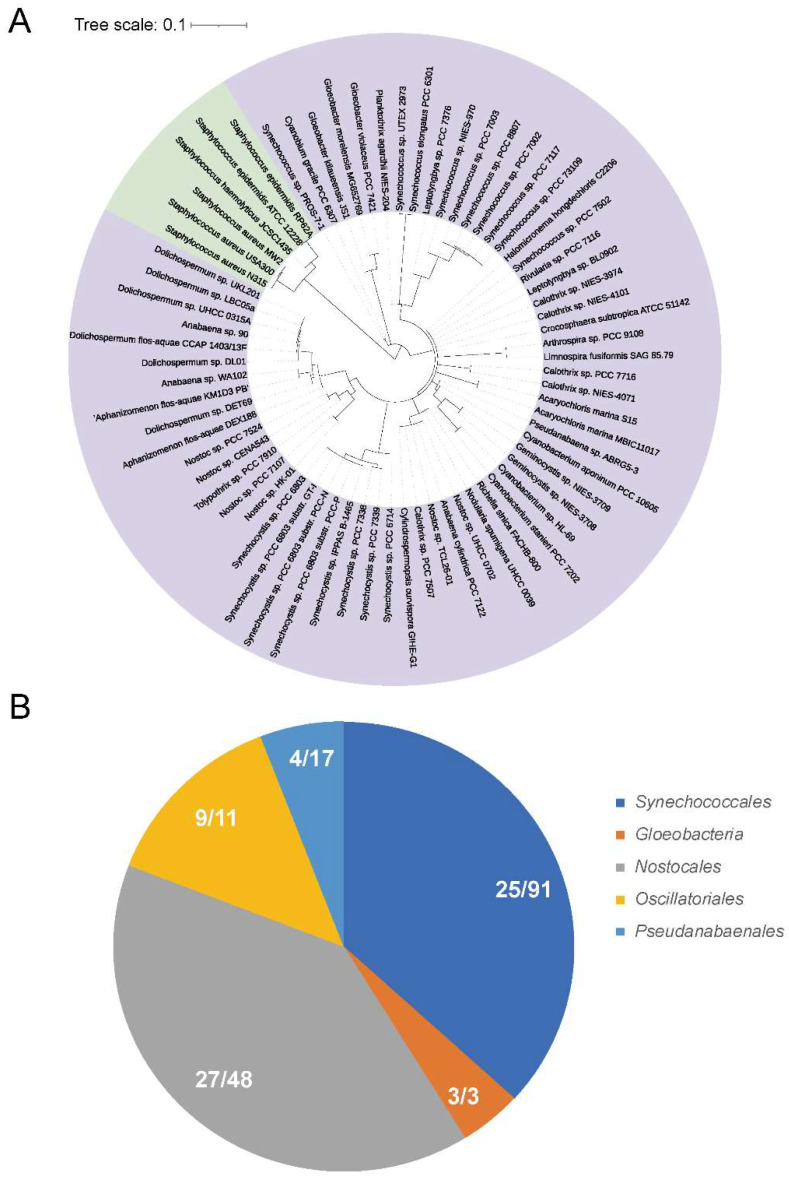
Phylogenetic analysis of PerR-encoding genes in the sequenced cyanobacteria genomes. (**A**) Phylogenetic tree of PerRs in cyanobacteria. A total of 68 probable PerRs were found in 198 cyanobacteria genomes. The representative proteins were labeled with name of species. The PerR queries were listed in Table S3. PerRs from *Staphylococcus epidermidis*, *Staphylococcus haemolyticus* and *Staphylococcus aureus* in Table S3 were used as the outgroup. (**B**) The distribution of PerR-encoding genes in cyanobacteria genomes. In total, 68 predicted PerR-encoding genes were detected among 64 cyanobacteria genomes, including 25 *Synechococcales*, 27 *Nostocales*, 3 *Gloeobacteria*, 9 *Oscillatoriales*, and 4 *Pseudanabaenalles*.

To investigate the distribution of PerR in cyanobacteria, we performed a BLASTsearch of the 198 cyanobacteria genomes (downloaded from the NCBI database on 17 December 2021) with the queries (Table S3) to find PerR candidates (Figure 1). *PerR* genes were identified using phylogenetic tree analysis (Figure 1A). In total, 68 PerR-encoding genes were distributed among 64 cyanobacteria genomes (Table S4). The cyanobacteria PerRs were distributed among five orders, including 25 *Synechococcales*, 27 *Nostocales*, 3 *Gloeobacteria*, 9 *Oscillatoriales*, and 4 *Pseudanabaenales* (Figure 1B). In *Gloeobacteria*, which was believed to be the early diverging lineage of cyanobacteria, all three of the published genomes within this order were encoded as *perR*. Furthermore, the proportions of species that contained *perR* in *Oscillatoriales* and *Nostocales* were 81.8% and 56.3%, respectively. For *Synechococcales*, the proportion was 27.5%, even though the total number of *PerR* genes was 25. For *Pseudanabaenales*, the proportion was only 23.5%.

### 3.2. PerR Deletion Increases the Tolerance of PCC7002 to High H_2_S_n_

**Figure 2:**
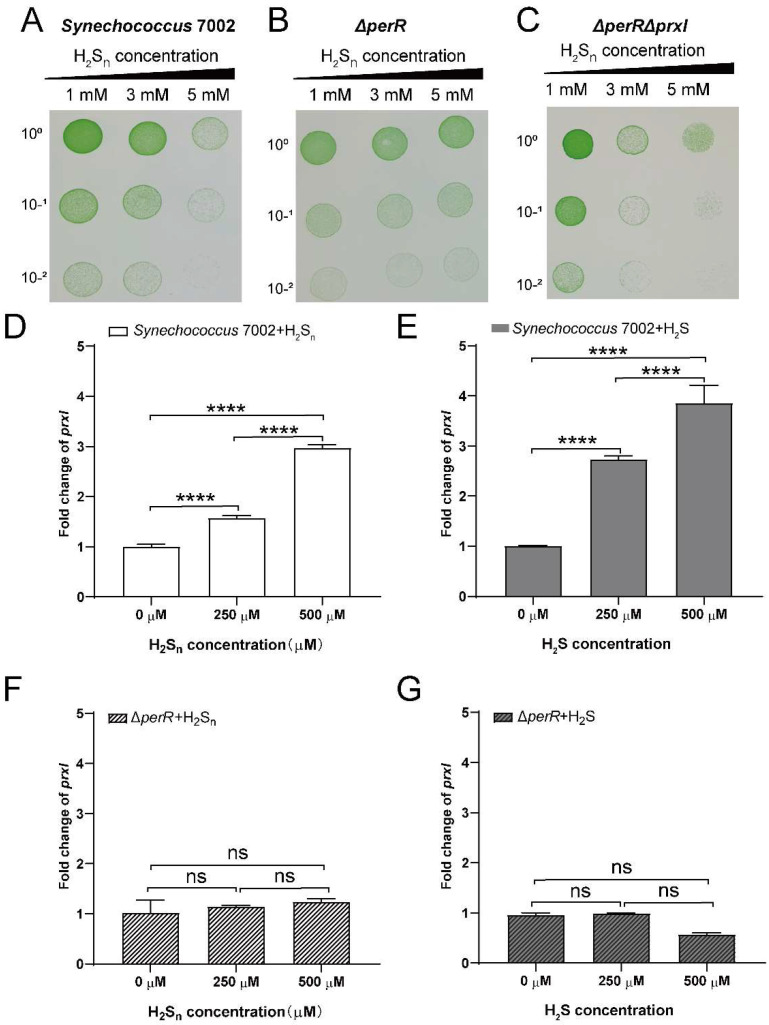
PerR decreases the tolerance of PCC7002 to H_2_S_n_. The deletion of *perR* (PCC7002Δ*perR*) (**B**) increased H_2_S_n_ tolerance, while the double deletion of *perR* and *prxI* (PCC7002Δ*prxI*Δ*perR*) (**C**) decreased H_2_S_n_ tolerance compared with the wild–type (**A**). PCC7002, PCC7002Δ*perR*, and PCC7002Δ*prxI*Δ*perR* cells at log phase with an OD_730 nm_ of 1 were treated with 1, 3, and 5 mM H_2_S_n_, at 30 °C, and 50 μmol photons m^−2^·s^−1^ illumination for 6 h. Then, cells were diluted with A^+^ medium to 10^0^, 10^−1^, and 10^−2^, and plated onto the A^+^ solid medium and cultured for 7 days, at 30 °C, and 50 μmol photons·m^−2^·s^−1^ illumination. The tolerance of PCC7002Δ*perR* and PCC7002Δ*prxI*Δ*perR* to H_2_S_n_ was opposite to that of the wild-type. The expression of *prxI* was largely upregulated by H_2_S_n_ (**D**) and H_2_S (**E**) in PCC7002, while H_2_S_n_ (**F**) and H_2_S (**G**) showed little influence on its expression in PCC7002Δ*perR*. PCC7002 and PCC7002Δ*perR* cells at log phase were induced by H_2_S_n_ and H_2_S with concentrations of 250 µM and 500 µM for 3 h, and the expression of *prxI* was measured. The *Y*-axis is the fold change in *prxI* calculated by relative quantitative qPCR, based on the 2^−ΔΔCT^ method, with *rnp* as the reference gene. All data are averages from three samples with standard deviations (error bars). The experiment was repeated at least three times. ****, *p* < 0.0001; ns, not significant (paired *t* test).

To investigate the effect of PerR on the tolerance of PCC7002 to sulfane sulfur, we constructed a single-deletion strain PCC7002Δ*perR*, and double-deletion strain PCC7002Δ*prxI*Δ*perR* by homologous recombination (Figure S1). The mutation was verified by PCR (Figure S1A). Then, the tolerance of PCC7002, PCC7002Δ*perR*, and PCC7002Δ*prxI*Δ*perR* to sulfane sulfur was tested (Figure 2). PCC7002Δ*perR* grew better than the wild-type after induction with 5 mM H_2_S_n_, indicating that the deletion of *perR* increased tolerance (Figure 2A,B). However, the double-deletion mutant (PCC7002Δ*prxI*Δ*perR*) showed decreased tolerance to H_2_S_n_, and growth inhibition was apparent after induction with 3 mM H_2_S_n_ (Figure 2C). These results indicated that PerR and PrxI were all involved in H_2_S_n_ tolerance of PCC7002.

### 3.3. PerR Senses H_2_S_n_ and Regulates the Expression of prxI

PerR acts as a transcriptional repressor in the regulation of *prxI* expression, as qPCR analysis showed that the transcript level of *prxI* was upregulated ~100-fold in PCC7002Δ*perR* compared with PCC7002 (Figure S1B). Furthermore, the expression levels of *prxI* were analyzed in PCC7002 and PCC7002Δ*perR* after induction with H_2_S_n_ and H_2_S to verify whether PerR is involved in the regulation of H_2_S_n_ metabolism. The expression of *prxI* increased 1.5-fold following 250 µM H_2_S_n_ induction and 3-fold following 500 µM H_2_S_n_ induction (Figure 2D); this effect was concentration dependent. H_2_S induction could also increase *prxI* expression by 2.5-fold at concentrations of 250 µM and 4-fold at concentrations of 500 µM (Figure 2E). However, neither H_2_S nor H_2_S*_n_* could induce the expression of *prxI* in PCC7002Δ*perR* (Figure 2F,G), which was different from the wild-type. Based on the above results, we deduced that PerR played a critical role in H_2_S_n_ sensing, thus regulating the expression of *prxI*.

**Figure 3:**
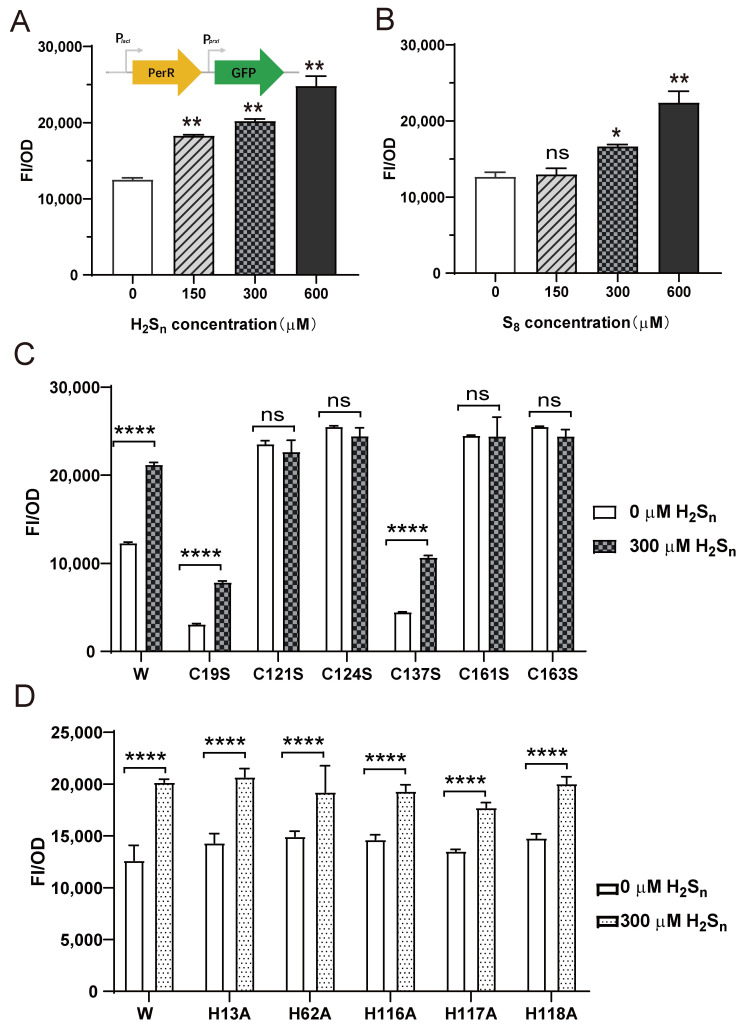
The effect of H_2_S_n_ and S_8_ on the PerR-repressed reporter. (**A**) Schematic representation of the test plasmid (pBBR-*perR*-P*prxI*-*egfp*). The expression of *egfp* was initiated by the *prxI* promoter (P*_prxI_*), and the interaction between PerR and P*_prxI_*, which was associated with the inducers that affected GFP fluorescence. H_2_S_n_ (**A**) and S_8_ (**B**) induction increased the intensity of GFP fluorescence in *E. coli* BL21 (pBBR-*perR*-P*prxI*-*egfp*). (**C**) The mutation of Cys affected the function of PerR in *E. coli* BL21 (pBBR-*perR*-P*prxI*-*egfp*). C19S, C121S, C124S, C137S, C161S, and C163S represented single mutations of Cys to Ser in PerR. (**D**) The mutation of His did not affect the function of PerR in *E. coli* BL21 (pBBR-*perR*-P*prxI*-*egfp*). H13A, H62A, H116A, H117A and H118A represented single mutations of His to Ala in PerR. FI/OD represents the fluorescence intensity of per OD cells. All data are averages from three samples with standard deviations (error bars). The experiment was repeated at least three times. *, *p* < 0.1; **, *p* < 0.01; ****, *p* < 0.0001; ns, not significant (paired *t* test).

Meanwhile, a PerR-repressed reporter system in *E. coli* BL21 was constructed to further assess the effect of H_2_S_n_ on *prxI* expression regulated by PerR (Figure 3). In the reporter system, the *perR* gene is controlled by the *lacI* promoter (PlacI), and the *egfp* gene is controlled by the *prxI* promoter (P*_prxI_*) (Figure 3A). When the expression of PerR was induced by IPTG, GFP fluorescence decreased significantly, indicating that the expressed PerR could act on the *prxI* promoter and inhibit the expression of GFP (Figure S2). Having verified that PerR acts on the promoter of *prxI* to inhibit its expression, the effects of H_2_S_n_ and S_8_ were tested. H_2_S_n_ induction caused an increase in fluorescence intensity (Figure 2A), and S_8_, another form of sulfane sulfur, had a similar effect (Figure 2B).

To test the critical role of Cys residues in PerR, all six Cys residues (Cys^19^, Cys^121^, Cys^123^, Cys^137^, Cys^160^, and Cys^163^) were individually mutated to Ser. The mutation of Cys^19^ and Cys^137^ (C19S and C137S) resulted in decreased fluorescence intensity but did not affect their response to H_2_S_n_. Cys^121^, Cys^124^, Cys^160^, and Cys^163^ were important components of the Cys_4_:Zn^2+^ site, and their mutation to Ser (C121S, C124S, C160S, and C163S) resulted in increased fluorescence intensities compared with the wild-type, indicating the inactivation of PerR (Figure 3C). As a result, PerR no longer acted on the *prxI* promoter to inhibit the expression of *egfp*, and it no longer responded to H_2_S_n_ induction. The mutation of His had no effect on PerR (Figure 3D), indicating H_2_S_n_ did not act on the Fe^2+^/Mn^2+^ site. We concluded that the expression of *prxI* was regulated by PerR, and the induction of S_8_ and H_2_S_n_ enhanced *prxI* expression by acting on PerR. Meanwhile, the Cys^121^, Cys^124^, Cys^160^, and Cys^163^ residues of PerR played crucial roles in H_2_S_n_ sensing.

### 3.4. Sulfane Sulfur Acts on the Cys_4_:Zn^2+^ Site of PerR

**Figure 4:**
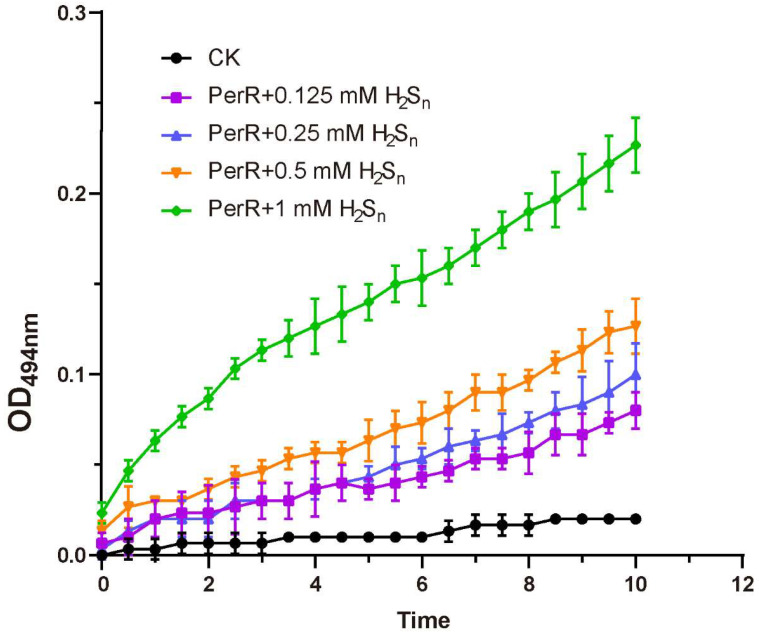
H_2_S_n_ caused dissociation of zinc ions from PerR. Oxidation of the Cys_4_:Zn^2+^ site by H_2_S_n_ led to Zn^2+^ release. H_2_S_n_ was incubated with 100 µM PerR, and formation of the Zn^2+^-PAR complex was continuously monitored by measuring the absorbance at 494 nm for 10 min. All data are averages from three samples with standard deviations (error bars). The experiment was repeated at least three times.

Furthermore, we measured the rate at which Zn^2+^ ions were released from PerR:Zn in the presence of H_2_S_n_ (Figure 4). An amount of 1 µM PerR contains about 0.125 µM Zn, 0.033 µM Fe and 0.006 µM Mn, as detected by ICP-MS. As the previous result (Figure 3D) confirmed that H_2_S_n_ did not act on the Fe^2+^/Mn^2+^ site, the effect on Cys_4_:Zn^2+^ site was monitored here. The formation of the colored Zn^2+^-PAR complex, whose absorption maximum was observed at 494 nm, was used to monitor the release of Zn^2+^. Thus, the Zn^2+^ release result showed that all selected concentrations of H_2_S_n_ induced the release of Zn^2+^. Based on the above results, we deduced the mechanism by which H_2_S_n_ acts on PerR, which involves H_2_S_n_ acting on the Cys_4_:Zn^2+^ site to dissociate Zn^2+^ from the active site, thus destroying the normal function of PerR.

**Figure 5:**
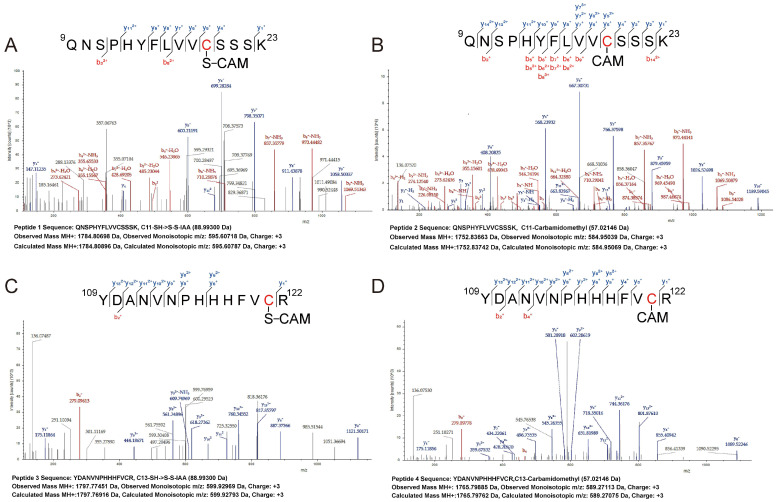
H_2_S_n_ acted on the cysteines of PerR. (**A**) The Cys^19^–SSH group was blocked by IAM in the peptide 1 from H_2_S_n_–treated PerR. (**B**) The Cys^19^–SH group was blocked by IAM in the peptide 2 from DTT-treated PerR. (**C**) The Cys^121^–SSH group was blocked by IAM in the peptide 3 from H_2_S_n_-treated PerR. (**D**) The Cys^121^–SH group was blocked by IAM in the peptide 4 from DTT–treated PerR. The purified PerR (5 mg/mL) was treated with 1 mM H_2_S_n_ and 1 mM DTT for 30 min, at 25 °C. After denaturing and incubating with IAM, the reacted protein was digested by trypsin. The generated peptides were detected by LTQ-Orbitrap tandem mass spectrometry.

Finally, we explored the mechanism by which H_2_S_n_ acts on the Cys_4_:Zn^2+^ site by LTQ-Orbitrap tandem mass spectrometry (Figure 5). Cys^19^-SH of Peptide 1 in H_2_S_n_-treated PerR was modified to Cys^19^-SSH (Figure 5A), while Cys^19^-SH of Peptide 2 in DTT-treated PerR was directly modified by acetamide (CAM) (Figure 5B). Similarly, Cys^121^-SH of Peptide 3 in H_2_S_n_-treated PerR was modified to Cys^121^-SSH (Figure 5C), while Cys^121^-SH of Peptide 4 in DTT-treated PerR was also directly modified by acetamide (CAM) (Figure 5D). Among these, Cys^121^ was an important constituent of the Cys_4_:Zn^2+^ site, thus indicating that H_2_S_n_ acts on Cys^121^ to inhibit the activity of PerR. Notably, Cys^19^ was also modified by H_2_S_n_, although it was not a component of the Cys_4_:Zn^2+^ site. In summary, the persulfide modification of Cys^121^ in the Cys_4_:Zn^2+^ site by H_2_S_n_ was the mechanism that affected PerR activity.

## 4. Discussion

**Figure 6:**
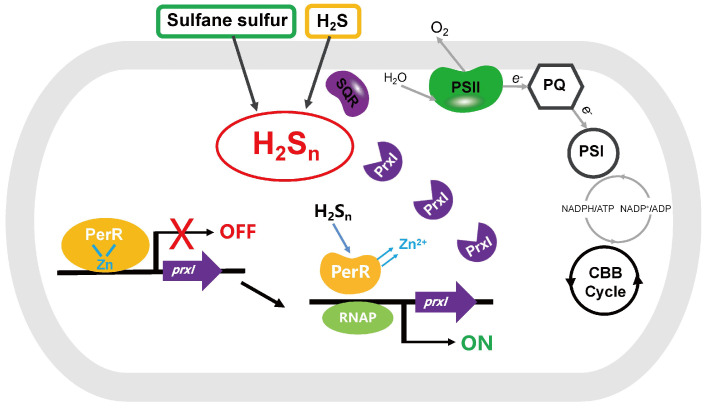
PerR senses H_2_S_n_ and regulates the expression of *prxI* in PCC7002. H_2_S_n_ induces the expression of *prxI* via PerR. PerR binds to Zn^2+^ and acts on the promoter of *prxI* to inhibit its expression, a process that can be disinhibited by H_2_S_n_. H_2_S_n_ acts on the Cys4:Zn^2+^ site of PerR to relieve Zn^2+^, destroying the zinc finger structure. C^121^-SH in the Cys4:Zn^2+^ site is modified by H_2_S_n_ and forms C^121^-SSH.

The data from our study revealed that PerR senses H_2_S_n_ and regulates the expression of *prxI* (Figure 6). The deletion of *perR* increased the tolerance for H_2_S_n_ in PCC7002 (Figure 2B), although the enhanced tolerance was not observed in the dual mutant PCC7002Δ*perR*Δ*prxI* (Figure 2C), indicating that PerR functioned by acting on PrxI. Meanwhile, the induction effect of H_2_S_n_ on *prxI* transcription levels was only observed in the presence of PerR (Figure 2), a result which was further confirmed by the PerR-repressed reporter system (Figure 3), indicating that PerR acted on the promoter region to inhibit *prxI* expression. H_2_S had similar effect with H_2_S_n_ on *prxI* transcription levels (Figure 2B). This effect may be caused by H_2_S_n_, which derive from H_2_S solution prepared from NaHS, as considerable levels of H_2_S_n_ may present [44]. Meanwhile, H_2_S may be converted to H_2_S_n_ by SQR [24]. Certainly, H_2_S may also act on PerR with a new mechanism directly, which needs to be further verified. Furthermore, H_2_S_n_ acted on the Cys^4^:Zn^2+^ site to release Zn^2+^, thus removing the inhibition (Figure 4) and allowing *prxI* to be expressed in large quantities to clear the excess sulfane sulfur. H_2_S_n_ modified Cys^121^ to form Cys^121^–SSH (Figure 5), destroying the structure of the Cys^4^:Zn^2+^ site and causing the release of Zn^2+^. Cys^19^ could also be modified by H_2_S_n_. Thus, H_2_S_n_ acted on the zinc figure structure of PerR, which represents a new type of mechanism for sulfane sulfur sensing.

Zinc-binding proteins are among the most abundant transcriptional regulators in eukaryotes, harboring at least one common motif, the zinc finger, which contributes to proper protein structure and function [58,59]. Zinc finger proteins are also found in prokaryotic genomes, such as *Bacillus* PerR [39] and *Synechococcus* PerR [9]. Zinc-binding proteins display variable secondary structures and vast functional diversity, and can be classified into three classes based on their distinct structural properties: Cys_2_His_2_ (C2H2) zinc finger proteins (Class I), Cys_4_ (C4) zinc finger proteins (Class II), and Cys_6_ (C6) zinc finger proteins (Class III). Class I proteins are often referred to as the classical zinc finger [60]. Class II proteins contain four cysteine residues bound to one zinc atom [61], whereas Class III proteins contain six cysteine residues bound to two zinc atoms [62]. Thus, *Synechococcus* PerR belongs to Class II, and this is the first report of a zinc-binding protein being involved in sulfane sulfur sensing.

OxyR and PerR are two representative regulators that can sense both H_2_O_2_ and H_2_S_n_. In total, 68 PerR proteins were identified among 198 sequenced cyanobacteria genomes (Figure 5), whereas only 9 OxyR proteins were identified (Table S5) [30], indicating that PerR may be the key player in cyanobacteria. Furthermore, PerR is a transcriptional inhibitor, whereas OxyR is a transcriptional activator, and their sensing mechanisms for H_2_O_2_ and H_2_S_n_ are quite different. The exact mechanism for the OxyR sensing of H_2_O_2_ is still under debate. The formation of a disulfide bond between Cys^199^ and Cys^208^ or the oxidization of Cys^199^ to C^199^-SOH in *E. coli* are two of the proposed mechanisms [63,64,65]. For H_2_S_n_ sensing, the Cys^199^ of *E. coli* OxyR is modified to Cys^199^-SSH [30]. *Bacillus* PerR senses H_2_O_2_ by metal-catalyzed oxidation [38], where one oxygen atom is incorporated into histidine 37 or histidine 91, which coordinates the bound Fe^2+^. Cysteines in the Cys_4_:Zn^2+^ site may also be oxidized by H_2_O_2_ [39]. Our results revealed that Cys^121^ in the Cys_4_:Zn^2+^ site of *Synechococcus* PerR could be modified by H_2_S_n_ to form Cys^121^-SSH (Figure 4), releasing one zinc atom and destabilizing the structure. Normally, PerR and OxyR do not exist in the same microbial strain, but there are some exceptions [66,67]. In cyanobacteria, PerR and OxyR did not coexist (Tables S4 and S5). Thus, although PerR and OxyR are considered functionally complementary, the mechanisms by which they function are different.

In addition to OxyR and PerR, two-component systems play an important role in H_2_O_2_ signal transduction in cyanobacteria [68]. A microarray-based study in *Synechocystis* PCC6803 revealed that His kinases (Hiks), namely, Hik33, Hik34, Hik16, and Hik42, are involved in the expression of a large number of H_2_O_2_-inducible genes [10]. Among the four Hiks, Hik33 was the main contributor and was responsible for the regulation of more H_2_O_2_-inducible genes than PerR. Furthermore, the response of *Synechocystis* to H_2_O_2_ treatment also relied on Group 2 sigma factors, namely, SigB and SigD [69]. The lack of Group 2 sigma factors meant that the strain was unable to sustain its growth under oxidative stress. Taken together, the signaling of H_2_O_2_-induced oxidative stress is based on the coordinated action of several regulators and dedicated alternative sigma factors. Whether these regulators participate in sulfane sulfur sensing requires further investigation.

The distribution of PerR proteins in cyanobacteria was also investigated. *Gloeobacterales* are early-branching photosynthetic cyanobacteria that are used as model species to study the physiology of early oxygenic phototrophs [70]. *Gloeobacterales* contain reduced photosystems that lack thylakoids and a circadian clock. However, our results revealed that all three species with published genomes within this order encoded *perR*, which may offer insight into the important role of PerR in primitive cyanobacteria and the evolution of oxygenic photosynthesis. Meanwhile, 81.8% of the species in *Oscillatoriales* and 56.3% of those in *Nostocales* contained PerR. *Oscillatoriales* and *Nostocales* are bloom-forming cyanobacteria that dominate among the cyanobacterial biomass of shallow polymictic eutrophic lakes [71]. The high proportion of PerR proteins among the two orders may provide insight into the survival strategies of cyanobacteria in hypoxic and sulfidic environments.

The finding that PerR senses H_2_S_n_ in cyanobacteria is significant. First, cyanobacteria have to tolerate the accumulation of sulfane sulfur in living environments. In Proterozoic oceans [2] and modern oxygen minimum zones [72], the environments in which cyanobacteria thrive are anoxic and sulfidic, and as a result, sulfane sulfur might accumulate. In cyanobacteria mats, a typical habitat for these microorganisms, the cyanobacteria are intermittently exposed to sulfane sulfur [73]. Although cyanobacteria can perform sulfur respiration and provide ATP for growth under dark and anoxic conditions by reducing sulfane sulfur [74], excess sulfane sulfur is fatal to cells [23]. Therefore, PerR sensing of H_2_S_n_ provides the opportunity for cyanobacteria to activate the expression of metabolic genes in time to scavenge excess sulfane sulfur, thus ensuring survival in such environments. Second, cyanobacteria perform anoxygenic photosynthesis under low-O_2_ and sulfidic conditions, using H_2_S as the electron donor [8,75]. In addition, cyanobacteria could produce some sulfur-containing histidine such as ergothioneine and ovothiols, which can also be used as electron donors [76,77]. As a result, sulfane sulfur was generated during the process of H_2_S oxidation by SQR during anoxygenic photosynthesis. Sulfane sulfur is a signal that participates in the regulation of physiology and critical gene expression in photosynthesis [24]. The PerR sensing of H_2_S_n_ may help cyanobacteria to maintain normal signal transduction and photosynthesis. Third, a previous study reported that the composition and stability of the photosynthetic machinery and the cell division process were affected by the overexpression of PerR [78], indicating that the effect of sulfane sulfur on the function of PerR may also affect the above process. Thus, the association between PerR, sulfane sulfur, photosynthesis, and cell division provides a new perspective on the significance of the PerR sensing of sulfane sulfur. In brief, the ability of PerR to sense H_2_S_n_ may ensure that cyanobacteria respond to intracellular and extracellular sulfane sulfur in a timely manner, allowing them to maintain normal photosynthesis and cell division, and adapt to environmental conditions.

## 5. Conclusions

In summary, we showed that PerR in PCC7002 senses H_2_S_n_ and regulates the expression of *prxI*. PCC7002 was able to respond in a timely manner to excess H_2_S_n_ in the environment with the help of PerR, enhancing its tolerance. H_2_S_n_ modified Cys^121^ of PerR to form Cys^121^-SSH, thus releasing Zn^2+^ from the Cys_4_:Zn^2+^ site, revealing a new mechanism of sulfane sulfur sensing in cyanobacteria. This is also the first report of a zinc-binding protein that participates in sulfane sulfur sensing. Our findings offer new insight into the mechanism of sulfane sulfur sensing and provide a new perspective for understanding the adaptation mechanism of cyanobacteria in anaerobic and sulfidic environments.

## Data Availability

Data are contained within the article and supplementary material.

## Supplementary Materials

The following supporting information can be downloaded at: https://www.mdpi.com/article/10.3390/antiox12020423/s1; Figure S1. The deletion of *perR* was verified by PCR and its effect on the transcriptional level of *prxI*; Figure S2. The expressed PerR could act on the *prxI* promoter and inhibit the expression of GFP; Table S1. Strains and plasmids used in this study; Table S2. Primers used in this study; Table S3. The queries used in the phylogenetic analysis of PerR; Table S4. The information of PerRs in cyanobacteria; Table S5. The OxyRs in cyanobacteria.
