# Supplementary material for: The Transcriptional Repressor PerR Senses Sulfane Sulfur by Cysteine Persulfidation at the Structural Zn2+ Site in Synechococcus sp. PCC7002
Source: Antioxidants (Basel). 2023 Feb 9;12(2):423. doi: 10.3390/antiox12020423 (PMC9952342; doi:10.3390/antiox12020423)
Supplement: Supplementary file 1 [file antioxidants-12-00423-s001.zip › 3 PerR SI 20230203.pdf]

Supplementary Information for

The transcriptional repressor PerR senses sulfane sulfur by cysteine persulfidation at the structural Zn^{2+} site in *Synechococcus* sp. PCC7002

Daixi Liu[1,2,3], Hui Song[1,3*], Yuanning Li[1,3], Ranran Huang[1,3], Hongyue Liu[1,3], Kunxian Tang[4], Nianzhi Jiao[1,3] and Jihua Liu[1,3]

[1]Institute of Marine Science and Technology, Shandong University, Qingdao, People's Republic of China

[2]School of pharmaceutical sciences, Shandong University, Jinan, People's Republic of China

[3]Joint Lab for Ocean Research and Education at Dalhousie University, Shandong University and Xiamen University

[4]Third Institute of Oceanography, Ministry of Natural Resources, Xiamen, People's Republic of China

* Hui Song

Email: 201799900009@sdu.edu.cn

This file includes:

Figure S1. The deletion of *perR* was verified by PCR and its effect on the transcriptional level of *prxI*.

Figure S2. The expressed PerR could act on the *prxI* promoter and inhibit the expression of GFP.

Table S1. Strains and plasmids used in this study

Table S2. Primers used in this study

Table S3. The queries used in the phylogenetic analysis of PerR

Table S4. The information of PerRs in cyanobacteria.

Table S5. The OxyRs in cyanobacteria.

Supplemental figures

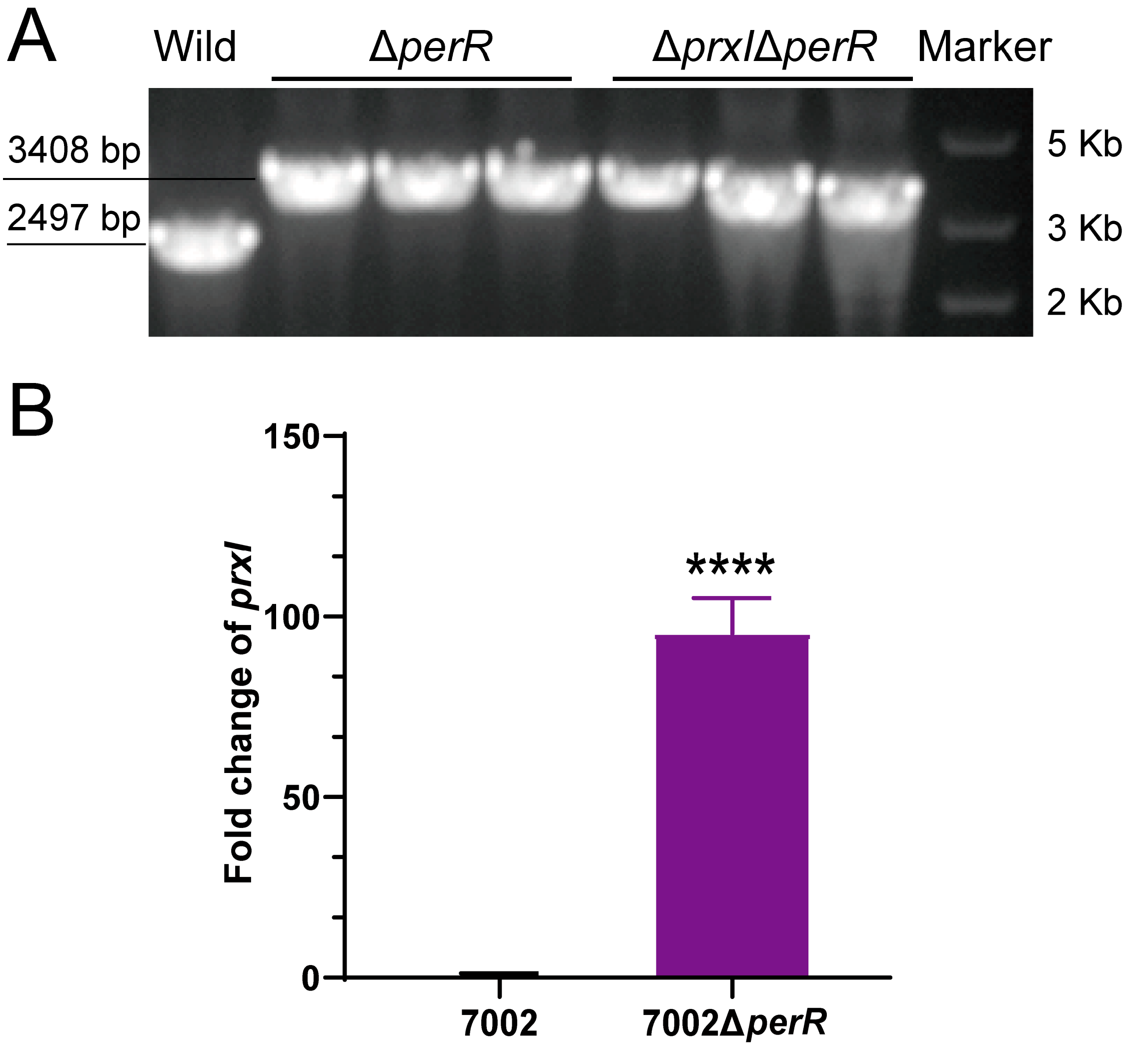

Figure S1. The deletion of *perR* was verified by PCR and its effect on the transcriptional level of *prxI.* **(A)** A 3408 bp fragment was generated while the *perR* gene was replaced by the chloramphenicol resistance cartridge. **(B)** PerR inhibited the expression of *prxI* in PCC7002. The transcriptional level of *prxI* in PCC7002Δ*perR* upregulated about 100-fold than that of the wild type verified by qPCR. ****, $p < 0.0001$.

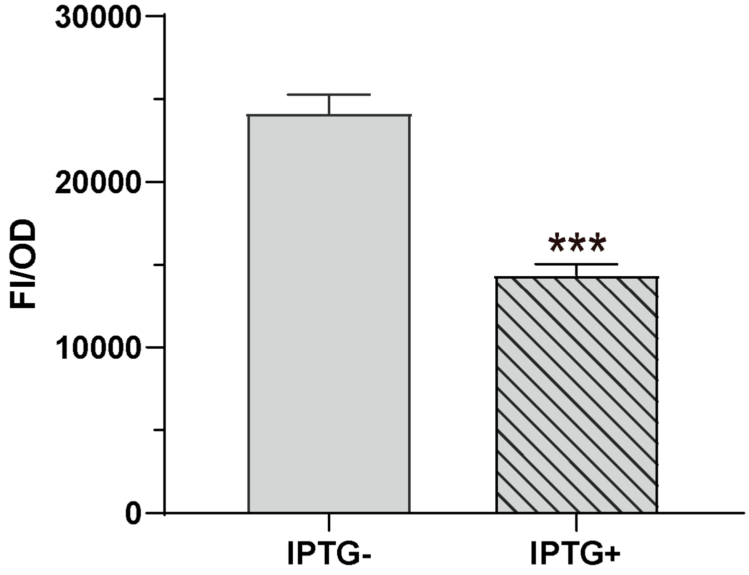

Figure S2. The expressed PerR could act on the *prxI* promoter and inhibit the expression of GFP. The expression of *perR* downregulated the fluorescence of GFP in *E. coli* BL21 containing the plasmid pBBR-*perR*-P_{prxI}-*egfp*. IPTG was used to induce PerR expression, which inhibited the expression of *egfp*. ***, $p < 0.001$.

Table S1 Strains and plasmids used in this study

Strain or plasmid	Description/characteristic	Source/reference
**Strains**		
PCC7002	Wilde type	This study
PCC7002Δ*perR*	PCC7002 with *perR* deletion	This study
	perR (Accession: ACA99824.1)	
PCC7002Δ*perR*Δ*prxI*	PCC7002 with *perR* and *prxI* deletion	This study
	prxI (Accession: WP_012306421.1)	
E. coli DH5α	Cloning strain	Novagen
E. coli BL21(DE3)	Cloning strain	Novagen
**Plasmids**		
pMal-C2X	expression vector	0ur lab
pMal-*perR*	pMal-C2X containing *perR*	This study
pJET1.2-Blunt	Cloning vector	Thermo Fisher
pET30	Cloning vector	0ur lab
pET30-*perR*	pET30 containing *perR*	This study
pJET-*perR*-del	*perR* complementation vector	This study
pBBR1MCS-2	Expression vector	This study
pBBR-P_{lacI}-PerR-P_{prxI}-eGFP	*prxI* promoter activity reporter with PerR	This study

Table S2 Primers used in this study

Primer name	Sequence
perR-del-1	AGCACACATCCCAACAACAGC
perR-del-2	CCAACTTAATCGCCTTGCAGCAGAGGGTAACAGTGTGG TCGGT
perR-del-3	ACCGACCACACTGTTACCCTCTGCTGCAAGGCGATTAA GTTGG
perR-del-4	GTGGGGATTGGTGTGGCTTCATACGACAGGTTTCCCGA CTGG
perR-del-5	CCAGTCGGGAAACCTGTCGTATGAAGCCACACCAATCC CCAC
perR-del-6	CATTTGTGTAGCGTGAATCAGG
pet30-*perR*-F	TAAGAAGGAGATATACATTTGATTTCACCTTGGCCCAT TCG
pet30-*perR*-R	TGGTGGTGGTGGTGCTCGAGAGCACGGCAGTCCGCAC
prxI-qPCR-F	AACGGTGAATTTACCCGCAA
prxI-qPCR-R	CCCTTGATGTAAGCCAGCAT
PerR-prxI-gfp-1 *gfp* (Accession: AAG29478.1)	CCGGCTCGTATGTTGTGTGGAATTGATTTCACCTTGGCC CATTC
PerR-prxI-gfp-2	TTAAGCACGGCAGTCCGCACA
PerR-prxI-gfp-3	TGTGCGGACTGCCGTGCTTAACAAAGCCCGAAAGGAA GCTGAG

PerR-prxI-gfp-4	GCGCTTAATGCGCCGCTACA
PerR-prxI-gfp-5	TGTAGCGGCGCATTAAGCGCGAAAGCCTTAGAAACGACCG
PerR-prxI-gfp-6	CTAGTATTTCTCCTCTTTCTCTAGATGTGGACCTCTTGGATAGTGT
PerR-prxI-gfp-7	TCTAGAGAAAGAGGAGAAATACTAGATGAGTAAAGGAGAAGAACTTTTC
PerR-prxI-gfp-8	CTTACAATTTCCATTCGCCATTCATTATTTGTATAGTTCATACATGCCAT
PerR-prxI-gfp-F	TTCCACACAACATACGAGCCGG
PerR-prxI-gfp-R	TGAATGGCGAATGGAAATTGTAAG
PerR C19S-F	GTCGTCTCTTCTTCCTCCAAAACCATGTCCCAAGC
PerR C19S-R	TTGGAGGAAGAAGAGACGACAAGGAAATAATGGGGGCTAT
PerR C121S-F	TTCGTCTCTCGAGACTGCGGGGCCATCCAAGAT
PerR C121S-R	CCGCAGTCTCGAGAGACGAAATGGTGGTGCGGATTAACATT
PerR C124S-F	CGAGACTCTGGGGCCATCCAAGATCTGCCTTG
PerR C124S-R	TGGATGGCCCCAGAGTCTCGACAGACGAAATGGTGGTG
PerR C137S-F	TTTAGCTCTTTTGATTTGGGTCAGTTTGCGACGCAAGTTAC
PerR C137S-R	ACCCAAATCAAAAGAGCTAAAGGCTTGCCAAGGCAGATCTTG

PerR C160S-F	GGACTTTCTGCGGACTGCCGTGCTTAATCGA
PerR C160S-R	ACGGCAGTCCGCAGAAAGTCCCTTAACGGTTACTTCATAGCT
PerR C163S-F	GCGGACTCTCGTGCTTAATCGAGCACCACCACC
PerR C163S-R	CTCGATTAAGCACGAGAGTCCGCACAAAGTCCCTTAACGGTTA

Table S3 The queries used in the phylogenetic analysis of PerR

Accession number	Name	Species
AKL85385.1	Transcriptional regulator PerR	*Bacillus atrophaeus*
P71086.1	Peroxide operon regulator	*Bacillus subtilis*
Q97FU2.1	Transcriptional regulator PerR	*Clostridium acetobutylicum*
Q57083.1	HTH-type transcriptional regulator PerR	*Escherichia coli*
Q2G282.1	Peroxide-responsive repressor PerR	*Staphylococcus aureus*
Q5HN74.1	Peroxide-responsive repressor PerR	*Staphylococcus epidermidis*
Q8CNQ7.1	Peroxide-responsive repressor PerR	*Staphylococcus epidermidis*
Q49YQ6.1	Peroxide-responsive repressor PerR	*Staphylococcus saprophyticus*
Q4L7G4.1	Peroxide-responsive repressor PerR	*Staphylococcus haemolyticus*
Q99T18.1	Peroxide-responsive repressor PerR	*Staphylococcus aureus*
Q7A4T8.1	Peroxide-responsive repressor PerR	*Staphylococcus aureus*
Q5HER3.1	Peroxide-responsive repressor PerR	*Staphylococcus aureus*
Q6GFJ6.1	Peroxide-responsive repressor PerR	*Staphylococcus aureus*
Q7A0J4.1	Peroxide-responsive repressor PerR	*Staphylococcus aureus*
Q2FFN4.1	Peroxide-responsive repressor PerR	*Staphylococcus aureus*
Q2YU25.1	Peroxide-responsive repressor PerR	*Staphylococcus aureus*
Q6G873.1	Peroxide-responsive repressor PerR	*Staphylococcus aureus*
Q9RQL3.1	Peroxide-responsive repressor PerR	*Staphylococcus aureus*

Table S5 The OxyRs in cyanobacteria.

Accession number	Name	Species
WP_110149640.1	hydrogen peroxide-inducible genes activator	*Nostoc sp. 3335mG*
WP_110149354.1	hydrogen peroxide-inducible genes activator	*Nostoc sp. 3335m*
PWL20063.1	DNA-binding transcriptional regulator OxyR	*Synechococcus sp. XM-24*
OWY61394.1	DNA-binding transcriptional regulator OxyR	*cyanobacterium TDX16*
WP_211737531.1	hydrogen peroxide-inducible genes activator	*Leptolyngbya sp. 15MV*
MBV5256979.1	hydrogen peroxide-inducible genes activator	*Synechococcus moorigangaii CMS01*
WP_068790708.1	hydrogen peroxide-inducible genes activator	*Phormidium willei*
MBC7758490.1	LysR family transcriptional regulator	*Phormidesmis sp. FL-bin-119*
MBY0493784.1	LysR family transcriptional regulator	*Cyanobacteria bacterium*
